# Predictors and clinical outcomes of permanent pacemaker implantation after transcatheter aortic valve implantation

**DOI:** 10.1186/s12872-024-04101-9

**Published:** 2024-08-24

**Authors:** Bing-Ying Chen, Ting-Feng Huang, Xin-Da Jiang, Xiao-Yan Ding, Xiao-Fen Zhou

**Affiliations:** 1 Shengli Clinical Medical College of Fujian Medical University, Fuzhou, 350001 Fujian China; 2 The Fourth Department of Intensive Care Unit, Fuzhou University Affiliated Provincial Hospital, Fuzhou, 350001 Fujian China; 3 Fujian Provincial Key Laboratory of Emergency Medicine, Fuzhou, 350001 Fujian People’s Republic of China; 4 Fujian Provincial Key Laboratory of Critical Care Medicine, Fuzhou, 350001 Fujian People’s Republic of China

**Keywords:** Permanent pacemaker, Procedural duration, Right bundle branch block, Transcatheter aortic valve implantation

## Abstract

**Objective:**

This study aimed to identify the incidence, risk factors, and outcomes of permanent pacemaker (PPM) implantation after transcatheter aortic valve implantation (TAVI) procedures.

**Methods:**

A retrospective analysis was conducted on 70 patients who underwent TAVI at the Department of Cardiology, Fujian Provincial Hospital, from January 2018 to March 2022. Based on whether a new PPM was implanted after TAVI, all patients were divided into two groups: NEW PPM and NO PPM. Baseline characteristics and clinical data were compared between the two groups. Univariate analysis was used to analyze different variables between the two groups. A binary logistic regression analysis was used to evaluate independent correlates for PPM implantation after TAVI.

**Results:**

The mean age of the 70 patients was 73.1 ± 8.8 years. The incidence of PPM implantation was 17.1%. Patients with diabetes and chronic kidney disease were more likely to require PPM (50% vs. 20.7%, *p* = 0.042, 25% vs. 5.2%, *p* = 0.042). Our study did not identify any significant differences in the incidence of electrocardiographic conduction disturbances except for the previous right bundle branch block (RBBB) (NO PPM 6.9% vs. NEW PPM 33.3%, *p* < 0.05). We found that prosthesis size, implantation depth, procedural duration, and length of hospital and intensive care unit (ICU) stays were comparable between the two groups. The leading independent predictors of PPM implantation were previous RBBB (odds ratio 10.129, *p* = 0.034).

**Conclusion:**

The previous RBBB was the leading independent predictor of PPM implantation. New PPM was not associated with significantly new-onset left BBB, extended post-procedure hospitalization, ICU stay, or procedural duration.

**Graphical Abstract:**

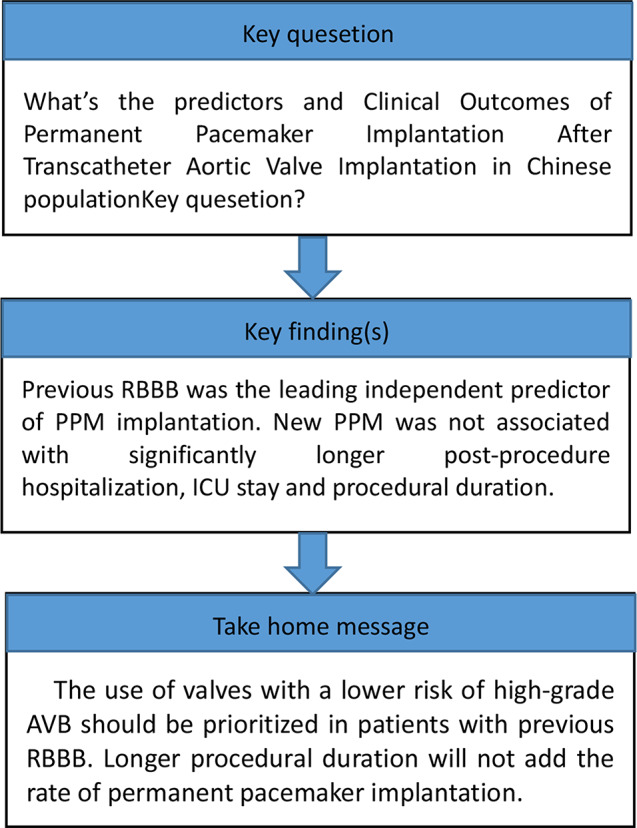

## Introduction

Over the past decade, transcatheter aortic valve implantation (TAVI) has become an inoperable treatment option. Since the first implantation by Prof. Cribier and his team in 2002, which received European conformity approval in the mid-2000s, the technology has spread worldwide. TAVI is considered a well-established therapy for patients with severe aortic valve disease who are deemed at high risk for surgical aortic valve replacement (SAVR) [1]. With advancements in TAVI technology and operators’ experience, TAVI effectiveness has been confirmed in lower-risk patients [2, 3]. However, despite rapid use, expanded indications, and improved technology, new conduction disorders after TAVI requiring pacemaker therapy remain a common postoperative complication [4]. Several studies have found that subsequent permanent pacemaker (PPM) implantation due to a high-degree atrioventricular block (AVB) is the most common complication after TAVI [5, 6]. Few studies have evaluated new PPM after TAVI in a Chinese population. However, studies on the effects of procedural duration on PPM implantation are lacking. Additionally, studies on the length of hospital and intensive care unit (ICU) stays in patients with new PPM after TAVI are limited. Current Chinese research on post-TAVI PPM implantation is predominantly characterized by small-scale, single-center studies, which often yield variable and sometimes contradictory results.For instance, Fang Du et al. identified a higher likelihood of PPM implantation in patients with pre-existing right bundle branch block (RBBB) [7]. However, Jiaqi Zhang et al. did not confirm the influence of pre-existing RBBB on PPM placement [8]. This discrepancy underscores the variability in findings across different studies and highlights the need for further research to elucidate the factors influencing post-TAVI PPM implantation in the Chinese population.We aim to expand our center’s study cohort to further investigate the factors influencing the rate of PPM implantation following TAVI in the Chinese population. By integrating data from multiple centers, we can obtain a more accurate and comprehensive understanding of the real-world scenarios of PPM implantation post-TAVI among Chinese patients.

Consequently, this study aimed to evaluate the incidence, risk factors, and outcomes of PPM implantation after TAVI, particularly in electrocardiographic parameters and echocardiographic characteristics, procedural characteristics, and length of stay at a hospital in a Chinese population.

## Patients and methods

### Patient population and study design

A retrospective analysis was conducted on 70 patients who received TAVI with the self-expandable venus A valve (Venus Med Tech Inc., Hangzhou, China) at the Department of Cardiology of the Fujian Provincial Hospital between January 2018 and March 2022. The study protocol was approved on June 30th, 2023, by the Fujian Provincial Hospital Human Research Ethics Committee (approval number: K2023-06-015). Considering the retrospective nature of this study, the need for informed consent was waived and approved by the Fujian Provincial Hospital Human Research Ethics Committee. All patients who underwent TAVI were deemed at medium- or high-risk for SAVR by the local cardiothoracic surgery team.

**Fig. 1:**
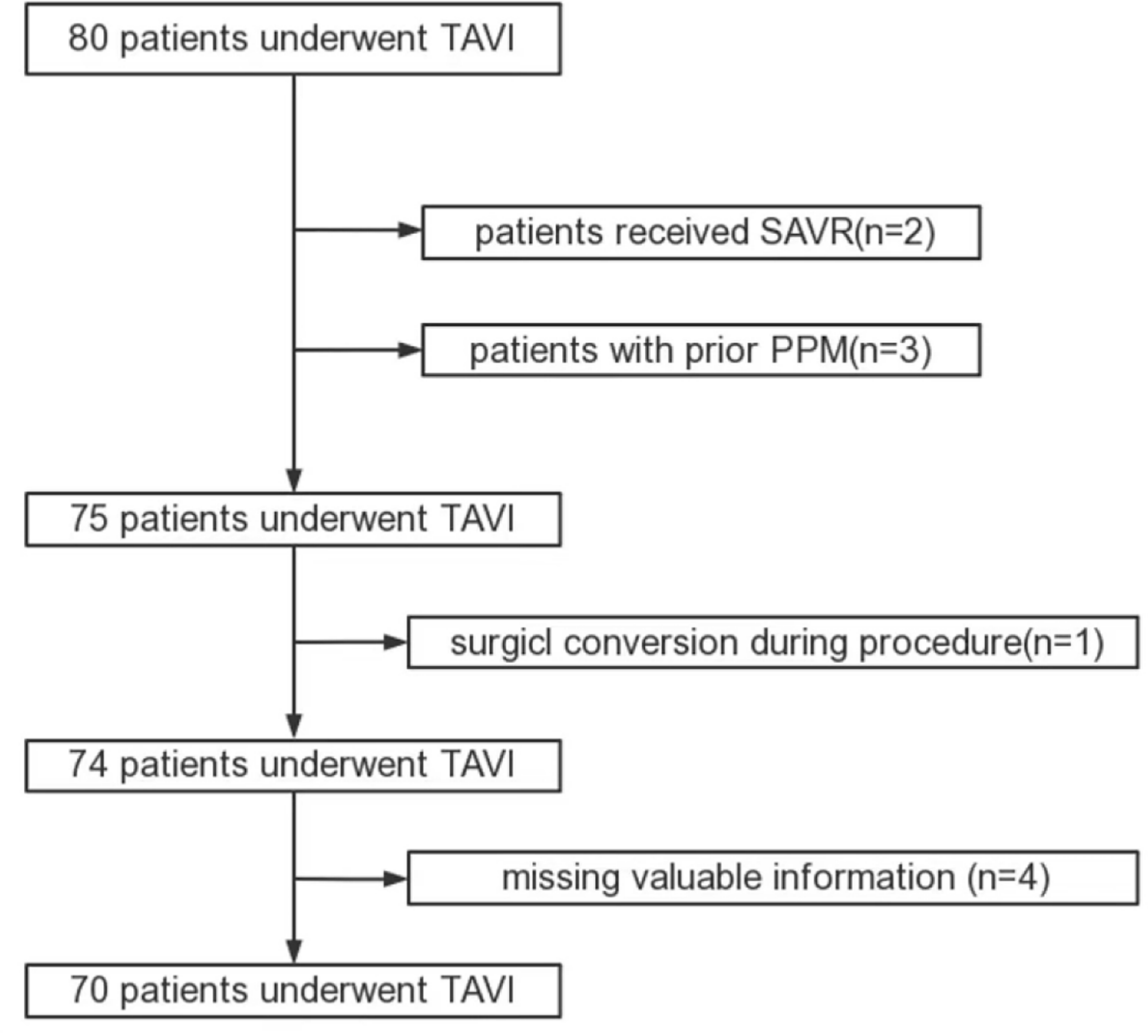
Study flowchart

The initial sample size was 80. Patients who had received SAVR (*n* = 2) or were previously treated with PPM (*n* = 3) were excluded. Additionally, one patient was excluded because of surgical conversion during the procedure (*n* = 1). Similarly, four patients with missing data for different clinical variables were excluded. Therefore, 70 patients were included in this study (Fig. 1). Based on whether a new PPM was implanted or not after TAVI, patients were divided into the NEW PPM group and the NO PPM group. Clinical data, such as electrocardiographic and echocardiographic data, were extracted retrospectively. Every patient underwent a baseline electrocardiogram and echocardiogram before TAVI. Baseline demographic information included age, gender, body mass index (BMI), history of hypertension, diabetes, and heart failure with different New York Heart Association functional classes (NYHA Class), atrial fibrillation, history of stroke, coronary artery disease, and chronic kidney disease. Echocardiographic variables included aortic valve peak velocity, aortic valve mean gradient, interventricular septum diastolic diameter, left ventricular end-diastolic diameter, left ventricular ejection fraction (LVEF), and bicuspid aortic valve.

### Statistical analysis

Baseline characteristics and clinical data were compared between the NEW PPM and NO PPM groups. Results are presented as n (%) for categorical data and mean ± standard deviation or median (interquartile range) for continuous variables according to variable distribution. Univariate analysis was done to analyze different variables between the two groups. A binary logistic regression analysis was used to evaluate independent correlates for PPM implantation after TAVI. Variables with a *p*-value less than 0.05 in the univariate analysis were subsequently included in the binary logistic regression model. Statistical Package for the Social Sciences software (version 19) was used to conduct the statistical analysis. A 2-sided *p*-value < 0.05 was considered statistically significant.

## Results

### Demographic and clinical characteristics

This study included 70 participants in the final analysis. All patients in our study were at intermediate or high surgical risk, with NYHA class III/IV, and none had a history of SAVR. Table 1 depicts the detailed clinical baseline characteristics of patients with or without PPM implantation. The mean age of the patients was 73.1 ± 8.8 years, and 68.6% of the study population was male. Complete AVB (100%) was an indication for PPM implantation after TAVI. The incidence of PPM implantation in our study was 17.1%. Baseline patient characteristics demonstrate that patients with diabetes were more likely to require PPM (50% vs. 20.7%, *p* = 0.042). Patients with chronic kidney disease were more likely to require PPM (25% vs. 5.2%, *p* = 0.042). The median time to PPM implantation after TAVI was four days among patients who underwent PPM implantation within 30 days of having TAVI.

**Table 1:** Baseline characteristics

	Total (*n* = 70)	NEW PPM (*n* = 12)	NO PPM (*n* = 58)	*p*-Value
Age (yrs)	73.1 ± 8.8	73.5 ± 7.1	73.1 ± 9.1	0.876
Male (%)	68.6	75.0	67.2	0.600
BMI (kg/m^2^)	23.4 ± 3.3	23.0 ± 3.8	23.4 ± 3.2	0.681
CAD (%)	34.3	25	36.2	0.460
CKD (%)	8.6	25	5.2	0.042*
Hypertension (%)	58.6	50	60.3	0.510
Diabetes (%)	25.7	50	20.7	0.042*
Prior stroke (%)	5.7	16.7	3.4	0.103
EuroScore	5(3,6)	4.5(3,6)	5(3.25,6)	0.441

CAD  = coronary artery disease; CKD = chronic kidney disease; PPM = permanent pacemaker; *= *p*˂0.05

### Electrocardiographic parameters and echocardiographic characteristics

Table 2 depicts the electrocardiographic change patterns in patients before and after TAVI. Our study did not find significant differences in the incidence of electrocardiographic conduction disturbances, including new-onset atrial fibrillation, new-onset first-degree AVB, new-onset right bundle branch block (RBBB), new-onset left BBB (LBBB), or any previous electrocardiographic conduction disturbances, except for the previous RBBB (NO PPM 6.9% vs. NEW PPM 33.3%, *p* = 0.017). Based on echocardiographic characteristics, patients with a higher LVEF were more likely to require PPM (60% vs. 57%, *p* = 0.045). Moreover, there were no differences between the two groups based on aortic valve peak velocity, aortic valve mean gradient, left ventricular end-diastolic, interventricular septum diastolic diameter, and the rate of bicuspid aortic valve, as illustrated in Table 2.

**Table 2:** Electrocardiographic parameters and echocardiographic characteristics

	NEW PPM (*n* = 12)	NO PPM (*n* = 58)	*p*-Value
Electrocardiographic parameters			
Before TAVR			
Sinus rhythm	83.3	87.7	0.683
atrial fibrillation	16.7	13.8	0.796
1°AVB	16.7	7.0	0.296
RBBB	33.3	6.9	0.017*
LBBB	0	8.6	0.999
After TAVR			
atrial fibrillation	0	5.3	0.999
RBBB	16.7	0	0.999
LBBB	16.8	26.3	0.485
3°AVB	100	0	1
Echocardiographic characteristics			
Before TAVR			
AV peak velocity (cm/s)	427.67 ± 157.794	467.84 ± 83.855	0.209
AV mean gradient (mmHg)	57.29 ± 31.95	55.76 ± 19.24	0.840
LVED (mm)	5.09 ± 0.81	5.11 ± 0.80	0.920
IVSd diameter (cm)	1.43 (1.30 1.54)	1.43 (1.26 1.56)	0.447
LVEF (%)	60 (58 65)	57 (52 60)	0.045*
BAV (%)	33.3	48.3	0.349

AV = aortic valve; AVB = atrioventricular block; BAV = bicuspid aortic valve; ECG = electrocardiogram; IVSd = interventricular septum diastolic diameter; LBBB = left bundle branch block; LV = left ventricular; LVED = left ventricular end-diastolic diameter; LVEF = left ventricular ejection fraction; PPM = permanent pacemaker; RBBB = right bundle branch block. *= *p*˂0.05

### Procedural characteristics and length of stays at the hospital

Table 3 depicts the procedural characteristics and length of hospital stays. The two groups could be compared based on prosthesis size, predilation balloon size, membrane length, implantation depth, procedural duration, and length of hospital and ICU stays.

**Table 3:** Procedural characteristics and length of stays at the hospital

	NEW PPM (*n* = 12)	NO PPM (*n* = 58)	*p*-Value
Procedural characteristics			
Prosthesis size (mm)	26 (24 26)	26 (26 29)	0.377
Predilation balloon size (mm)	22 (20 23)	22 (22 23)	0.446
Predilation balloon (%)	75	91.4	0.121
Membranous membrane length (mm)	9.50 (9.00 10.50)	9.25 (9.00 10.38)	0.943
Implantation depth (mm)	3 (1 5)	4 (1 4)	0.571
Overspeed stimulation (bpm)	180 (160 180)	180 (180 180)	0.872
Procedural duration (min)	140 (110 180)	165 (134.75 200.00)	0.278
stay			
Length of hospital stay (days)	19.50 (16.25 21.75)	17 (13 24)	0.282
Length of ICU stay (days)	1 (1 1)	1 (1 1)	0.637

PPM = permanent pacemaker, ICU = intensive care unit stay, *= *p*˂0.05

### Univariate and binomial logistic regression analysis of PPM after TAVR

Table 4 depicts that the main independent predictors of PPM implantation were previous RBBB (odds ratio 10.129, *p* = 0.034). Univariate analysis revealed that diabetes, chronic kidney disease, and LVEF were associated with PPM implantation. However, the association became statistically non-significant after the confounder adjustment.

**Table 4:** Univariate and binomial logistic regression analysis of PPM after TAVR

	Univariate	Binomial Logistic Regression	*p*-Value
	Odds Ratio	Odds Ratio	
Age	0.876	0.980	0.701
Gender	1.462	1.167	0.855
CKD	6.111	3.040	0.357
Prior RBBB	6.750	10.129	0.034*
Diabetes	3.833	4.351	0.073
LVEF	1.124	1.130	0.051

CKD = chronic kidney disease; RBBB = right bundle branch block.*= *p*˂0.05

## Discussion

TAVI is being increasingly utilized in treating patients with severe symptomatic aortic valve disease. Despite significant improvements in device technologies and implantation techniques, cardiac conduction disturbances that cause the new PPM implantation after TAVI remain a frequent complication. In this study, we analyzed clinical data, electrocardiographic parameters, echocardiographic characteristics, procedural characteristics, and length of hospital stays of 70 patients at the cardiovascular department of Fujian Provincial Hospital. Our study aimed to identify the incidence, risk factors, and outcomes of PPM implantation after TAVI to analyze the possible causes of new PPM implantation in the Chinese population.

Numerous studies have identified various ECG, imaging, and procedural risk factors for PPM in different ways. Factors, including the depth of implantation below the aortic valve annulus, prosthesis size, and the height of the membranous ventricular septum and left ventricular outflow tract, have received notable importance [9–12]. However, most of these studies were small-scale trials, potentially limiting their generalizability to the entire population. Therefore, this study also analyzed the electrocardiographic parameters and characteristics and procedural characteristics. However, no significant results were found among these factors. Additionally, we observed that the previous RBBB impacted the new PPM implantation after TAVI.

The indications for PPM may vary depending on the criteria of different centers, and they may not always follow current guidelines. The new PPM implantation was all due to the high degree of AVB in our study. In the TAVI procedure, most conduction disturbances occur in the acute period (periprocedural or within 24 h of the procedure) [13]. It is generally believed that TAVI causes two injuries to the conduction system, balloon dilation and valve implantation, resulting in a complete atrioventricular block [14]. In this study, the RBBB prevalence among patients reached approximately 11.4%, and new PPM is required in 50% of patients with previous RBBB. Previously, RBBB was the leading independent predictor of PPM implantation in patients who underwent TAVI. Several studies have found that baseline RBBB is probably the strongest and most consistent clinical predictor of PPM, which has been identified in over half of the studies evaluating multivariable predictors of PPM [15, 16]. Watanabe et al. identified 749 TAVI recipients and found that 13.6% of patients with previous RBBB exhibited higher rates of PPM implantation, which was similar to the study by Auffret et al., who studied 3,527 patients who received TAVI and found that 10.3% of patients with previous RBBB have higher rates of PPM implantation [15, 17]. Some other studies found a similar outcome [12, 16, 18]. Underlying degenerative conduction system disease may increase the susceptibility of the conduction system to injury in the course of TAVI [19]. Patients with previous RBBB are more likely to develop high-grade AVB due to the impaired conduction system. Mechanical et al. emphasized that when TAVI is used on the aortic valve, local edema and deterioration of the ventricular septum can harm atrioventricular conduction [18]. The proximity between the aortic valve and the conduction system explains the genesis of periprocedural conduction disturbances during TAVI. Waksman R et al. reported that the incidence of bradyarrhythmia and high-grade AVB increased when the LBBB was injured during valve deployment or aortic balloon valvuloplasty in patients with previous RBBB [20]. Considering the high rate of PPM implantation in patients with previous RBBB receiving a self-expanding valve, using valves with a lower risk of high-grade AVB should be prioritized to reduce the PPM implantation rate [15].

Conduction disturbances after TAVI include high-grade AVB and new-onset LBBB. Currently, there is no agreement on the LBBB effect on the new PPM implantation after TAVI. PPM rates of 5–14% have been reported at follow-up among patients with new-onset LBBB, many of which were associated with the progression toward high-grade AVB being the most frequent indication for PPM across studies [21–23]. The incidence of new-onset LBBB varies after TAVI. Several studies have reported that the rate of new-onset LBBB after TAVI ranges from 4 to 65%, depending on the valve type [24]. The two most commonly used valves are the self-expanding and balloon-expandable valves. However, new-onset LBBB in this study was not a risk factor for new PPM implantation. All patients in our center only received a self-expanding valve, which could explain the disparities in outcomes. However, the inclusion of transient LBBB in some studies and differences in measurement timing can all contribute to the difference.

There is no unified conclusion on the preoperative left ventricular ejection fraction implantation rate to the new PPM after TAVI. Herein, it was statistically different in the NO PPM and NEW PPM groups. However, the P-value was at the critical state, and the median was 57% and 60%, which was clinically non-significant. Therefore, further data analysis and research are still needed for this factor.

Several centers have analyzed the risk factors for PPM implantation after TAVI. However, most of them did not focus on the effect of procedural duration. In this study, the median procedural duration was 140 min in the NEW PPM group and 165 min in the NO PPM group, but a non-significant statistical difference was found. The longer duration was not associated with the new-onset PPM implantation in our study. No other study has reported the relevant content. The reasons for this phenomenon are as follows: (1) the procedural duration did not affect conduction tract damage. The reason is that the longer duration did not damage the conduction tract. (2) The procedural duration was steady in various centers. Possibly, there was a non-significant difference in the damage to the conduction tract when the duration was in a certain time range. However, this is only a unilateral conjecture, and the data did not support these conjectures.

Fadahunsi OO et al. found that PPM implantation was associated with a prolonged length of stay in the hospital and the ICU, which is inconsistent with our findings [25]. We analyzed the length of hospital and ICU stays with new PPM implantation and found no statistical difference from those without PPM implantation. Different geographical environments, indications for prolonged hospitalization length, and registries can influence the outcome. Fadahunsi OO et al.‘s study registry included all patients with aortic stenosis [25]. However, patients with aortic stenosis and aortic insufficiency were included in our study, which could explain the disparities in outcomes. The study did not mention discharge and ICU indications. Thus, comparing them was difficult.

### Study limitations

This is a small sample study of patients with TAVI at a single center, which did not include all centers or the total number of TAVI procedures performed in Fujian Province, China. The small sample size limits the statistics of the study. Additionally, this analysis had inherent restrictions related to the retrospective data analysis. There was no information on the type of self-expanding that might have allowed us to explore the reasons for the new PPM implantation. Moreover, the medications that could affect cardiac conduction were unspecified in this study. The method for determining implantation depth was not standardized and unavailable in this study. However, the depth of implantation may be the influencing factor for PPM. Some imaging findings associated with higher PPM implantation rates in many works of literature were not assessed. Despite the limitations of our study, we believe that it is representative and could provide valuable perspectives in identifying high-risk cases requiring PPM. As TAVI indications expand to younger and lower-risk patients with fewer additional risks and longer expected survival, TAVI is becoming more popular and accessible globally. Consequently, studies in different centers and regions are necessary to confirm the effect of PPM on clinical outcomes. Our findings favor an early intervention for postoperative patients who may require PPM.

## Conclusions

PPM was required within 30 days of TAVI in 17.1% of patients who did not previously undergo PPM. Previously, RBBB was the leading independent predictor of PPM implantation in patients who underwent TAVI. New PPM was not associated with significantly longer post-procedure hospitalization, ICU stay, or procedural duration. Considering the high rate of PPM implantation in patients with previous RBBB, valves with a lower risk of high-grade AVB should be prioritized to reduce the PPM implantation rate. More extensive studies with collaboration among departments are required to improve post-TAVI new PPM implantation rates and continue allowing TAVI to evolve as a safe and reproducible intervention.

## Data Availability

The data that support the findings of this study are available from Key Laboratory of Big Date Project of Fujian Province but restrictions apply to the availability of these data, which were used under license for the current study, and so are not publicly available. Data are however available from the corresponding authors upon reasonable request and with permission of Key Laboratory of Big Date Project of Fujian Province.

## Abbreviations

AF: Atrial Fibrillation
AVB: Atrioventricular Block
BMI: Body Mass Index
ICU: Intensive Care Unit
LBBB: Left Bundle Branch Block
NYHA Class: New York Heart Association functional classes
PPM: Permanent Pacemaker
RBBB: Right Bundle Branch Block
SAVR: Surgical Aortic Valve Replacement
TAVI: Transcatheter Aortic Valve Implantation
